# Skin Temperatures in Females Wearing a 2 mm Wetsuit during Surfing

**DOI:** 10.3390/sports7060145

**Published:** 2019-06-14

**Authors:** Mackenzie E. Warner, Jeff A. Nessler, Sean C. Newcomer

**Affiliations:** Department of Kinesiology, California State University, San Marcos, CA 92096-001, USA; kessl009@cougars.csusm.edu (M.E.W.); jnessler@csusm.edu (J.A.N.)

**Keywords:** thermal comfort, clothing design, clothing testing, sports ergonomics, physiology

## Abstract

The aim of this investigation was to examine regional skin temperatures in recreational female surfers’ wearing a 2 mm thick neoprene wetsuit while surfing and to compare these results to previously published data collected in males participating in an identical study. Female surfers (n = 27) engaged in surfing for at least 40 min while wearing a commercially available 2 mm full wetsuit. Skin temperature of eight different anatomical locations were measured with wireless iButton thermal sensors. Regional skin temperatures significantly differed (*p* < 0.001) across almost all anatomical regions. Furthermore, regional skin temperatures significantly decreased across time at all skin regions throughout an average surfing session (*p* < 0.001). The greatest reduction in skin temperature was observed in the lower leg (−5.4 °C). Females in the current study exhibited a significantly greater skin temperature decrease in the lower back (−15.2% vs. −10.8%, *p* = 0.022) and lower arm (−13.6% vs. −10.8%, *p* < 0.001) when compared to previous data published in males. Overall, results of the current study are consistent with data previously published on male recreational surfers. However, the current study provides preliminary evidence that the magnitude of change in skin temperature may differ between male and female recreational surfers at some anatomical locations.

## 1. Introduction

Participation in the sport of surfing has increased significantly over the past century and, as a result, surfers have exposed themselves to more extreme conditions in search of the perfect wave [1,2,3]. Many of these athletes surf in cold water and ambient air temperatures, which require them to wear wetsuits in an attempt to create a thermal barrier and maintain normothermia [4]. Wetsuits help to maintain body temperature by reducing convective heat loss [5], which can be three to five times greater in water than air [6]. For example, skin temperatures in triathletes during swimming are significantly higher while wearing a wetsuit compared to no wetsuit trials [7]. Convective heat loss is an important consideration for aquatic athletes, because it can lead to alterations in internal muscle temperature, which significantly impact performance [8,9,10,11,12].

Surfing presents a unique challenge to thermoregulation when compared to other aquatic sports because various regions of the body interact with the water at differing degrees across the surf session. During an average surf session, the recreational surfer spends only 4–8% of the time wave riding, while 44–58% and 28–42% is spent paddling and stationary in the water, respectively [1,2,13,14,15,16]. These behaviors lead to inconsistent rates of convective heat loss through both water and air. We recently reported that heat loss, as measured by skin temperature, is heterogeneously distributed across the body of recreational male surfers wearing 2 mm wetsuits during an average surf session [17]. The greatest reduction in skin temperature (−6.4 °C) was reported to occur in the distal portion of the leg [17], which interacts with water for a greater percentage of time relative to other regions of the body.

While regional heat losses have been demonstrated in male surfers, data suggest that males and females may respond differently to cold stress. Specifically, in response to cold stress, females generally exhibit a decrease in maximum heat production generated by either shivering or exercise [18], lower mean skin temperatures resulting in increased body heat debt [19,20], and accelerated rate of extremity cooling [21]. Potential mechanisms for these responses include lower total body mass, greater relative body fat percent [22], differences in body fat distribution [23], lower skeletal muscle mass [23,24], and decreased arterial flow to the hands and feet [21] compared to males. While there are physiological differences between the sexes, it remains unclear if heat loss across the body differs between females and males participating in surfing. Therefore, the aim of this investigation was to examine skin temperatures across the body of recreational female surfers’ wearing a 2 mm thick neoprene wetsuit while surfing. The primary hypothesis of the study was that the greatest decrease in skin temperature would occur in the lower leg, due to repeated immersion in the water. The secondary hypothesis of the study was that females would have overall greater heat loss when compared to previously reported data in males that participated in an identical research protocol.

## 2. Materials and Methods

### 2.1. Participants

Data were collected on twenty-seven adult female recreational surfers (age: 31.67 ± 1.52 years, height: 1.68 ± 0.01 m, body mass: 59.67 ± 1.20 kg, body mass index: 21.25 ± 0.38 kg/m^2^, surfing experience: 10.5 ± 1.22 years) with at least one year of surfing experience. Prior to data collection, participants completed both a questionnaire that gathered information about height, body mass, wetsuit preferences, board preferences, and personal demographics, and an informed written consent that was approved by the Institutional Review Board at California State University San Marcos (Protocol #1159522) in accordance to the Declaration of Helsinki.

### 2.2. Experimental Overview

Experimental procedures for this study have previously been described elsewhere from an identical study investigating skin temperatures of recreational male surfers wearing a wetsuit between the months of March and May [17]. Briefly, eight wireless iButton thermal sensors (type DS1921G; Maxim Integrated/Dallas Semiconductor Corp., San Jose, CA, USA) were attached to the skin of the calf, thigh, forearm, upper arm, chest, upper back, lower abdomen, and lower back with waterproof adhesive bandages (Nexcare^TM^ Tegaderm^TM^, St. Paul, MN, USA) and measured skin temperature at 1 min intervals [25,26]. Heart rate was also measured at 1 min intervals with a receiver (RCX5) secured to the wrist and a transmitter (T31) strapped across the chest (Polar Electro Inc., Kempele, Finland). Following instrumentation participants were provided a 2 mm full wetsuit (Hurley, Costa Mesa, CA, USA) to wear over all thermal sensors and the heart rate monitor. Surfers were asked not to wear a swimsuit or rash guard underneath their wetsuit. Participants were then instructed to engage in their usual surfing activities for at least 40 min. During the surf session, air temperature, water temperature, wave direction, wave interval, wave height, wind speed, and tide were obtained from the National Oceanic and Atmospheric Administration’s buoys (Surfline.com).

### 2.3. Data Analysis

Skin temperature data from the iButtons was downloaded using the OneWireViewer (Maxim Integrated/Dallas Semiconductor Corp., San Jose, CA, USA) Java^TM^ application at the completion of each surf session. Only skin temperature measurements recorded during the participant’s surf sessions were used for the analysis. The polarpersonaltrainer.com website was used to retrieve and downloaded heart rate data. Samples recorded as zero beats-per-minute were not used in the analysis.

### 2.4. Statistical Analyses

Skin temperature (iButtons) data from each anatomical region were evaluated for normality using the Shapiro–Wilk test and for differences in variance using Mauchley’s and Levene’s tests. Data at each time point for 6 anatomical regions were found to be normally distributed (Shapiro–Wilk, *p* > 0.05). Data for the chest and upper arm regions were not normally distributed for multiple time points. Following verification of homogeneity of variance, the data were analyzed using a mixed model analysis of variance (8 anatomical regions × 8 time points), where skin temperature data were reduced to 8 time points by averaging temperature across 5 min epochs during the first 40 min of data collection for each participant. While many participants surfed for durations longer than 40 min, the statistical analysis was truncated to 40 min to standardize time in the water and maximize sample size by including participants who chose to surf for only 40 min. A significant result for the main effect of anatomical region was followed up with separate Wilcoxon signed-rank test for sensor data, since a portion of the data sets were not normally distributed. A significant result for the main effect of time was followed up with Wilcoxon signed-rank tests comparing mean temperature for min 1–5 with min 36–40 for all 8 sensors. Finally, post hoc comparison of the interaction effect of anatomical region by time was performed by first calculating the percent change in temperature between min 1–5 and min 36–40 at each sensor location, and then comparing percent change pairwise among all 8 anatomical regions using Wilcoxon signed-rank tests. The Benjamini–Hochberg analysis was applied to all pairwise comparisons in order to control for false discovery rate [27]. Values are reported as mean ± standard error (SE).

In order to evaluate the hypothesis that females would experience greater heat loss compared to males, data from the current study were compared with data obtained in males using an identical protocol [17]. Percent change in temperature over 40 min was compared at each anatomical region between males (n = 46) and females (n = 27) using separate Mann–Whitney U tests. In addition, practical significance was evaluated by calculating Cohen’s *d* effect size for the percent change at each anatomical region.

## 3. Results

### 3.1. Surf Activity Profile and Environmental Conditions

Participants (n = 27) in the study surfed for an average of 63.3 ± 3.1 min and achieved an average and maximal heart rate of 131 ± 1 and 164 ± 3 beats per minute, respectively. Twenty-seven subjects surfed for at least 40 min, and 13 subjects surfed for more than 65 min. The average water temperature, wave size, wave interval, and wave direction were 14.6 ± 0.2 °C, 0.50 ± 0.02 m, 9.0 ± 1.0 s, and 242 ± 7°, respectively. In addition, average air temperatures, wind speed, and wind direction were 16.4 ± 0.5 °C, 2.7 ± 0.4 km/h, and 225 ± 15°, respectively.

### 3.2. Regional Skin Temperatures

**Figure 1:**
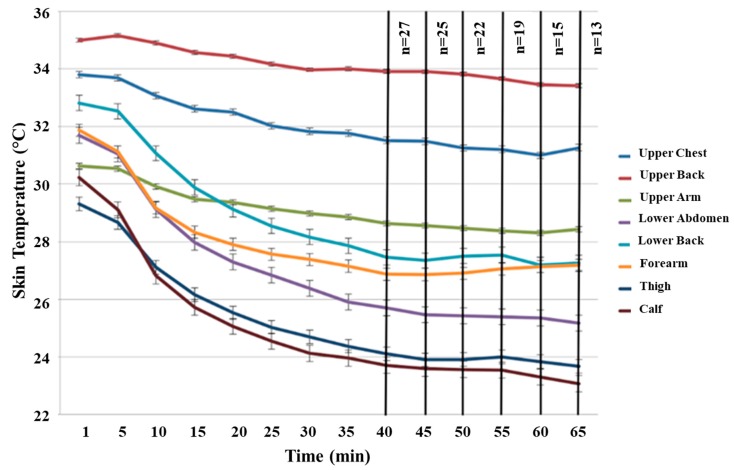
Average regional skin temperatures from 27 surf sessions of at least 40 min in duration (Mean ± SE). * Statistical analysis was performed at 40 min and skin temperatures were displayed up to 65 min.

Figure 1 represents average skin temperatures across a 65 min surf session at the eight anatomical regions. There was a significant difference between nearly all of the regions studied (main effect for anatomical region: *F*_7,45_ = 69.723, *p* < 0.001).

Specifically, post hoc comparison revealed significant differences in skin temperature between all anatomical regions except for the upper arm vs. lower back, lower back vs. forearm, and thigh vs. calf (Table 1).

### 3.3. Changes in Skin Temperatures

**Figure 2:**
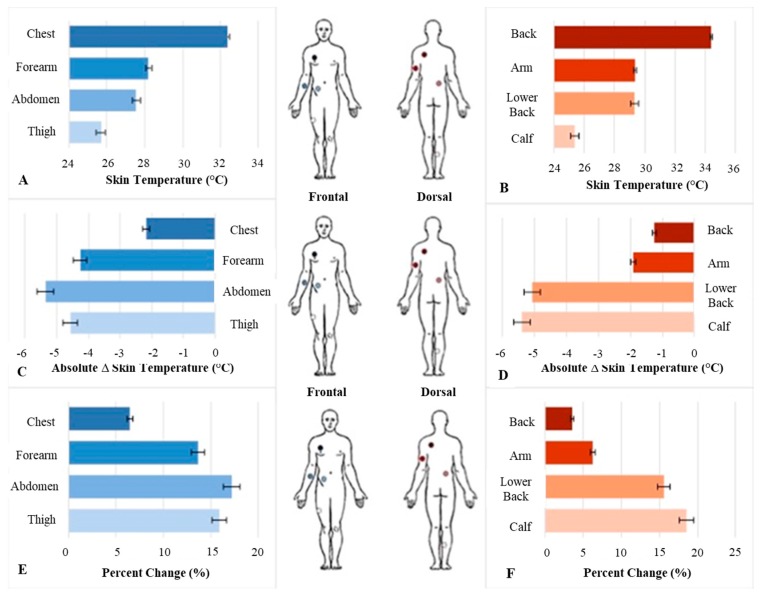
Frontal and dorsal average skin temperatures (**A**,**B**), absolute change in skin temperature (**C**,**D**), and percent change (**E**,**F**) across eight anatomical locations during twenty-seven recreational surf sessions. All data are expressed as mean ± SE.

Regional skin temperature significantly decreased across time during the surf sessions (main effect for time: *F*_7,45_ = 448.414, *p* < 0.001; Wilcoxon signed-rank test, *p* < 0.001). The average decrease in skin temperature for the first 40 minutes of the surf session ranged from 3.1% (back) to 18.4% (calf), with an average percent change of 12.15% ± 2.41% across all eight anatomical locations (Figure 2). There was also a significant time–sensor location interaction (*F*_7,45_ = 10.79, *p* < 0.001). This resulted in substantial variation in the magnitude of change in skin temperature across regions, with six noteworthy exceptions: chest vs. arm, lower abdomen vs. lower back, lower abdomen vs. thigh, lower abdomen vs. calf, low back vs. forearm, and lower back vs. thigh, (Table 1).

### 3.4. Sex Differences in Skin Temperature

Comparison of the current skin temperature data acquired in females, with previously published skin temperature data collected in males [17], suggested that the percent change in skin temperature after 40 min of recreational surfing in a 2 mm wetsuit was not significantly different in males versus females in the upper back, chest, upper arm, upper leg, or calf. However, females did exhibit a significantly greater decrease at the lower back compared to males (−15.2% vs. −10.8%, respectively, *p* = 0.022) [17]. Additionally, females exhibited a significantly greater decrease at the lower arm compared to males (−13.6% vs. −10.8%, respectively, *p* < 0.001) [17]. Likewise, the lower abdomen trended towards females displaying significantly greater decreases in skin temperature (*p* = 0.134). Calculation of Cohen’s *d* indicated that there was a large effect size at the lower arm, medium effect sizes at the lower back, and small effect sizes for the lower abdomen, upper arm and upper leg (Table 2).

## 4. Discussion

The increased participation of females surfing in cold water environments has made neoprene wetsuits a standard piece of surfing equipment. Interestingly, wetsuit manufactures currently have no data to inform female wetsuit design, since there is a paucity of research characterizing the impact that wetsuits have on thermoregulation in females. Therefore, we conducted a field study, which characterized the skin temperatures of female surfers across eight anatomical regions while wearing a 2 mm wetsuit during average surf sessions. The findings from this study demonstrate for the first time that regional skin temperatures of female recreational surfers significantly differ across most anatomical regions studied. Furthermore, skin temperatures significantly decreased across an average surf session. While the degree of decrease in skin temperatures varied between anatomical regions, these data show that the percent change in skin temperature was significantly different among almost all anatomical regions studied. The greatest decrease was observed in the lower leg (−18.8%), which equates to a −5.4 °C absolute change in skin temperature. These findings support our primary hypothesis that the greatest decrease in skin temperature would occur in the lower leg, which is likely attributed to its repeated immersion in cold water [17].

Analysis of previously collected data in males, compared to our current data, suggest that there may be subtle differences in skin temperature changes across a surf session between recreational male and female surfers wearing a 2 mm wetsuit (Table 2). While the magnitude of decrease varied across anatomical regions, percent change in skin temperature ranged from an average of −3.1% to −18.8% in females and −4.3% to −18.1% in males [17]. However, females in the current study had significantly greater decreases in lower back and lower arm skin temperature than what had previously been reported in males [17]. In addition, there was a trend (*p* = 0.134, Cohen’s *d* = 0.45) for females in the current study to have a greater decrease in abdominal skin temperature compared to males [17]. It is important to note that these differences in regional skin temperatures between sexes need to be interpreted with caution given the fact that environmental conditions between studies were not identical. Specifically, throughout the duration of the current study on females, average water temperature (14.6 ± 0.2 °C) and average air temperature (16.4 ± 0.5 °C) were colder than the average water temperature (16.0 ± 0.1 °C) and average air temperature (18.7 ± 0.4 °C) throughout the duration of the male study [17]. Since convective heat loss is three to five times greater in water than in the air [6], colder water temperatures may have played a role in the greater decrease in the percent change of skin temperature in females. In addition, average wave size (0.5 ± 0.02 m) in the current study was less than the average wave size in the male study (0.94 ± 0.03 m) [17]. Since the average wave size in the current study was less than that recorded during the study on males, females may have spent more time stationary than males, thus spending more time partially submerged in the water and less time paddling and wave riding. Therefore, the inability to control for environmental conditions between studies only provides preliminary evidence of sex differences in skin temperature during recreational surfing. Future studies will need to control for environmental factors to definitively determine if differences in skin temperatures exist between male and females participating in recreational surfing.

In conclusion, these findings demonstrate for the first time that the lower extremities have greater decreases in skin temperatures when compared to the upper extremities in females participating in recreational surfing while wearing a 2 mm full wetsuit. We have previously published similar findings in male surfers, with the greatest decrease in regional skin temperature also occurring in the lower body [17]. However, evidence from this study suggests that female surfers may experience greater decreases in percent change of some regional skin temperature locations throughout a surf session when compared to males. These findings are significant since reductions in skin temperatures of similar magnitude have been reported to negatively affect muscle function [8,9]. Specifically, decreases in lower extremity skin temperatures similar to our current findings in females have been associated with decreases in internal muscle temperature, which leads to reductions in average force production (−48%), maximal force production (−21%), power output (−32%), and take-off velocity during a vertical jump (−18%) [10,11,12]. Surf performance can be significantly affected by these reductions in skeletal muscle performance because surfers have to quickly transition from a prone paddling position to a standing and wave-riding position [28]. Consequently, the significant reduction in lower limb skin temperatures in the current study may greatly hinder surfing performance. Therefore, altering future female wetsuits through the alteration of neoprene thickness in the lower extremities may enhance performance by increasing skin temperature. In addition, since females may experience greater decreases in skin temperatures in some regions compared to males, neoprene distribution in wetsuits may need to be different for each sex. However, future studies that control for body composition and environmental factors that contribute to thermoregulation during surfing are required to definitively determine if differences exist between skin temperatures of men and women participating in recreational surfing.

## Figures and Tables

**Table 1:** *p*-values for pairwise comparisons of skin temperature at different anatomical locations (n = 27, 0–40 min). * Represents pairwise comparisons of skin temperature that did not achieve significance.

*P*-Values	Means								
	Thermistor	Chest	Back	Arm	Abdomen	Low Back	Forearm	Thigh	Calf
Percent change	Chest		*p* < 0.001	*p* < 0.001	*p* < 0.001	*p* < 0.001	*p* < 0.001	*p* < 0.001	*p* < 0.001
	Back	0.02		*p* < 0.001	*p* < 0.001	*p* < 0.001	*p* < 0.001	*p* < 0.001	*p* < 0.001
	Arm	0.886 *	0.010		0.001	0.486 *	0.011	*p* < 0.001	*p* < 0.001
	Low Abd	*p* < 0.001	*p* < 0.001	*p* < 0.001		*p* < 0.001	0.107 *	*p* < 0.001	*p* < 0.001
	Low Back	*p* < 0.001	*p* < 0.001	*p* < 0.001	0.037		0.097 *	*p* < 0.001	*p* < 0.001
	Forearm	*p* ≤ 0.001	*p* < 0.001	*p* < 0.001	*p* < 0.001	0.269 *		*p* < 0.001	*p* < 0.001
	Thigh	*p* < 0.001	*p* < 0.001	*p* < 0.001	0.068 *	0.28 *	0.020		0.387 *
	Calf	*p* < 0.001	*p* < 0.001	*p* < 0.001	0.220 *	0.004	*p* < 0.001	0.012	

**Table 2:** Effect size comparison of percent change in skin temperature across eight anatomical locations in men and women surfers during a 40 min session. All data are expressed as mean ± SE.

Location	% Change Male (n = 46)	% Change Female (n = 27)	Cohen’s *d*
Chest	5.90 ± 0.94	6.73 ± 1.49	0.117
Back	4.30 ± 0.79	3.08 ± 1.08	−0.222
Arm	6.14 ± 0.89	5.86 ± 1.42	−0.042
Low Abd	15.06 ± 0.87	17.53 ± 0.98	0.450
Low Back	10.76 ± 1.16	15.25 ± 1.22	0.628
Forearm	9.05 ± 0.70	13.60 ± 0.88	0.977
Thigh	14.52 ± 0.90	15.91 ± 0.94	0.252
Calf	18.08 ± 0.59	18.84 ± 0.82	0.185

## References

[1] Meir R.A., Lowdon B.J., Davie A.J. (1991). Heart rates and estimated energy expenditure during recreational surfing. J. Sci. Med. Sport.

[2] Loveless D.J., Minahan C. (2010). Two reliable protocols for assessing maximal-paddling performance in surfboard riders. J. Sports Sci..

[3] Moran K., Webber J. (2013). Surfing injuries requiring first aid in New Zealand, 2007–2012. Int. J. Aquat. Res. Ed..

[4] Naebe M., Robins N., Wang X., Collins P. (2013). Assessment of performance properties of wetsuits. J. Sports Eng. Technol..

[5] Wakabayashi H., Kaneda K., Sato D., Tochihara Y., Nomura T. (2008). Effect of non-uniform skin temperature on thermoregulatory response during water immersion. Eur. J. Appl. Physiol..

[6] Nimmo M. (2004). Exercise in the cold. J. Sports Sci..

[7] Groeller H. (1990). The Effects of Wearing a Wetsuit in the Thermoregulatory and Cardiovascular Responses Observed During a Triathlon in Elite Male Competitors.

[8] Bennett A.F. (1984). Thermal dependence of muscle function. Am. J. Physiol..

[9] Holewijn M., Heus R. (1992). Effects of temperature on eletromyogram and muscle function. Eur. J. Appl. Physiol..

[10] Sargent A.J. (1987). Effect of muscle temperature on leg extension force and short-term power output in humans. Eur. J. Appl. Physiol..

[11] Oksa J., Rintamaki H., Rissanen S. (1997). Muscle performance and eletromyogram activity of the lower leg muscles with different levels of cold exposure. Eur. J. Appl. Physiol..

[12] Bergh U., Ekblom B. (1979). Influence of musle temperature on maximal strength and power output in human skeletal muscles. Acta Physiol. Scand..

[13] Barlow M.J., Findlay M., Gresty K., Cooke C. (2014). Anthropometric variables and their relationship to performance and ability in male surfers. Eur. J. Sport Sci..

[14] Farley O., Harris N.K., Kilding A.E. (2012). Physiological demands of competitive surfing. J. Strength Cond. Res..

[15] LaLanne C.L., Cannady M.S., Moon J.F., Taylor D.L., Nessler J.A., Crocker G.H., Newcomer S.C. (2017). Characterization of activity and cardiovascular responses during surfing in recreational male surfers between the ages of 18–75 years old. J. Aging Phys. Act..

[16] Mendez-Villanueva A., Bishop D., Hamer P. (2006). Activity profile of world-class professional surfers during competition: A case study. J. Strength Cond. Res..

[17] Corona L.J., Simmons G., Nessler J.A., Newcomer S.C. (2018). Characterization of regional skin temperatures in recreational surfers wearing a 2 mm wetsuit. Ergonomics.

[18] Astrand I. (1960). Aerobic work capacity in men and women. Acta Physiol. Scand..

[19] Buskirk E.R., Thompson R.H., Whedon G.D. (1963). Metabolic response to cold air in men and women in relation to total body fat content. J. Appl. Physiol..

[20] Wyndham C.H., Morrison J.F., Williams C.G., Bredell G.A., Peter J., Vonrahden M.J., Holdsworth L.D., Vangraan C.H., Vanresburg A.J., Munro A. (1964). Physiological reactions to cold of Caucasian females. J. Appl. Physiol..

[21] Bollinger A., Schlumpf M. (1976). Finger blood flow in healthy subjects of different age and sex in patients with primary Raynaud’s Disease. Chir. Scand. Suppl..

[22] Durnin J.V., Womersley J. (1974). Body fat assessed from total body density and its estimation from skinfold thickness: Measurements on 481 men and women aged from 16–72 years. Br. J. Nutr..

[23] Abe T., Kearns C.F., Fukunaga T. (2003). Sex differences in whole body skeletal muscle mass measured by magnetic resonance imaging and its distribution in young Japanese adults. Br. J. Sports Med..

[24] Janssen L., Heymsfield S.B., Wang Z.M., Ross R. (2000). Skeletal muscle mass and distribution in 468 men and women aged 18–88 yr. J. Appl. Physiol..

[25] Van Marken Lichtenbelt W.D., Dannen H.A., Wouters L., Fronczek R., Raymann R.J., Severens N.M., Van Someren E.J. (2006). Evaluation of wireless determination of skin temperature using iButtons. Phys. Behav..

[26] Vickers M., Schwarzkopf L. (2016). A simple method to predict body temperature of small reptiles from environmental temperature. Ecol. Evol..

[27] Benjamini Y., Hochberg Y. (1995). Controlling the false discovery rate: A practical and powerful approach to multiple testing. J. R. Stat. Soc. Ser. B.

[28] Sheppard J.M., McNamara P., Osborne M., Andrews M., Oliveira Borges T., Walshe P., Chapman D.W. (2012). Association between anthropometry and upper-body strength qualities with sprint paddling performance in competitive wave surfers. J. Strength Cond. Res..

